# 5-(4-Pyrid­yl)-1,3,4-thia­diazole-2(3*H*)-thione

**DOI:** 10.1107/S1600536810052116

**Published:** 2010-12-18

**Authors:** Xu-Feng Liu, Xing-Hai Liu

**Affiliations:** a Department of Chemical Engineering, Ningbo University of Technology, Ningbo 315016, People’s Republic of China; b College of Chemical Engineering and Materials Science, Zhejiang University of Technology, Hangzhou 310014, People’s Republic of China

## Abstract

The title compound C_7_H_5_N_3_S_2_, occurs as the thione tautomer in the solid state; the dihedral angle between the pyridine and thia­diazole ring planes is 2.08 (6)°. In the crystal, mol­ecules are linked by N—H⋯N hydrogen bonds, generating *C*(8) chains propagating in [010].

## Related literature

For details of the synthesis, see: Song *et al.* (2005 ▶). For the biological activity of related compounds, see: Liu *et al.* (2007 ▶, 2009*a* ▶,*b* ▶,*c* ▶).
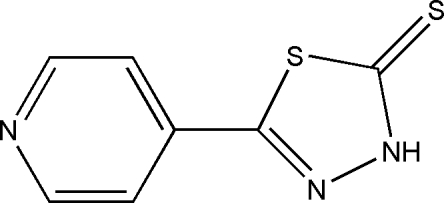


## Experimental

### 

#### Crystal data

C_7_H_5_N_3_S_2_
*M* *_r_* = 195.26
Monoclinic, 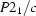

*a* = 7.837 (3) Å
*b* = 15.971 (5) Å
*c* = 6.694 (2) Å
β = 103.680 (4)°
*V* = 814.1 (5) Å^3^
*Z* = 4
Mo *K*α radiation
μ = 0.59 mm^−1^
*T* = 113 K
0.20 × 0.20 × 0.08 mm

#### Data collection

Rigaku Saturn CCD diffractometer
Absorption correction: multi-scan (*CrystalClear*; Rigaku/MSC, 2005 ▶) *T*_min_ = 0.891, *T*_max_ = 0.954
8141 measured reflections
1928 independent reflections
1642 reflections with *I* > 2σ(*I*)
*R*_int_ = 0.033

#### Refinement

*R*[*F* ^2^ > 2σ(*F* ^2^)] = 0.028
*wR*(*F* ^2^) = 0.076
*S* = 1.06
1928 reflections
113 parameters
1 restraint
H atoms treated by a mixture of independent and constrained refinement
Δρ_max_ = 0.44 e Å^−3^
Δρ_min_ = −0.21 e Å^−3^

### 

Data collection: *CrystalClear* (Rigaku/MSC, 2005 ▶); cell refinement: *CrystalClear*; data reduction: *CrystalClear*; program(s) used to solve structure: *SHELXS97* (Sheldrick, 2008 ▶); program(s) used to refine structure: *SHELXL97* (Sheldrick, 2008 ▶); molecular graphics: *SHELXTL* (Sheldrick, 2008 ▶); software used to prepare material for publication: *SHELXL97*.

## Supplementary Material

Crystal structure: contains datablocks global, I. DOI: 10.1107/S1600536810052116/hb5770sup1.cif

Structure factors: contains datablocks I. DOI: 10.1107/S1600536810052116/hb5770Isup2.hkl

Additional supplementary materials:  crystallographic information; 3D view; checkCIF report

## Figures and Tables

**Table 1:** Hydrogen-bond geometry (Å, °)

*D*—H⋯*A*	*D*—H	H⋯*A*	*D*⋯*A*	*D*—H⋯*A*
N1—H1⋯N3^i^	0.90 (1)	1.85 (1)	2.7395 (19)	169 (2)

Symmetry code: (i) 
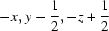
.

**Fig. 1.**
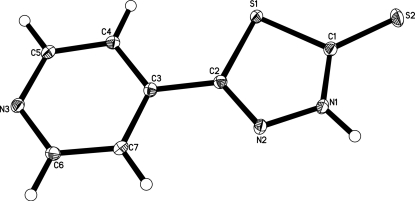
The molecular structure of (I). Displacement ellipsoids are drawn at the 30% probability level and H atoms are shown as small spheres of arbitrary radii.

**Fig. 2.**
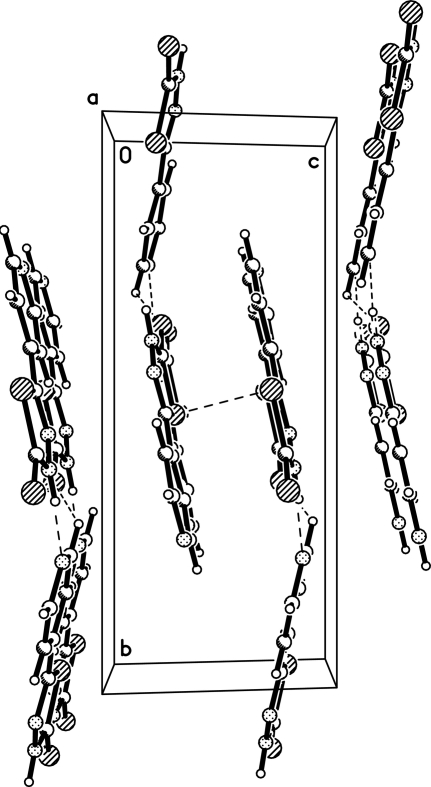
The crystal packing for (I).

## supplementary crystallographic information

### Comment

Thidiazoles had excellent biological activities, such as fungicide, KARI (Liu *et al.*, 2007, 2009*a*,*b*,*c*). Meanwhile, some nicotine structure are also exhibited good biological activity. In continuated our work, a thidiazoles derivatives had been synthesized. The strucuture was confirmed by X-ray crstallography.

Single-crystal X-ray diffraction analysis reveals that the title compound crystallizes in the monoclinic space group P2(1)/c. As shown in Fig. 1, the pyridine ring and the thiadiazole ring are nearly in the same plane [dihedral angle = 2.1 °]. As shown in Fig. 2, the crystal structure is stabilized by weak N—H···N intermolecular interactions.

### Experimental

Potassium hydroxide (0.11 mol) was dissolved in minimum amount of ethanol, and 4-nicotinehydrazide (0.1 mol) was added to it. The reaction mixture was cooled to 0–5 °C followed by dropwise addition of carbon disulfide (0.11 mol). After addition, the reaction mixture was stirred for 30 min to afford solid potassium dithiocarbazate salt. It was filtered, washed with EtOH, dried, and used as such for further reaction. Potassium dithiocarbazate salt (0.1 mol) was added slowly in small lots to conc sulfuric acid (2.5 times of salt) at 5 °C with constant stirring. The reaction mixture was stirred for 30 min, and the resulting viscous liquid was poured over crushed ice slowly. The solid obtained was filtered and washed and dried to get the title compound. The compound was recrystallized in DMF to yield colorless prisms.

### Refinement

All the H atoms were positioned geometrically (C—H = 0.93–0.97 Å) and refined as riding with *U*_iso_(H) = 1.2*U*_eq_(C) or 1.5*U*_eq_(methyl C).

### Crystal data

**Table:**

C_7_H_5_N_3_S_2_	*F*(000) = 400
*M**_r_* = 195.26	*D*_x_ = 1.593 Mg m^−^^3^
Monoclinic, *P*2_1_/*c*	Mo *K*α radiation, λ = 0.71073 Å
Hall symbol: -P 2ybc	Cell parameters from 2887 reflections
*a* = 7.837 (3) Å	θ = 2.6–27.9°
*b* = 15.971 (5) Å	µ = 0.59 mm^−^^1^
*c* = 6.694 (2) Å	*T* = 113 K
β = 103.680 (4)°	Prism, colorless
*V* = 814.1 (5) Å^3^	0.20 × 0.20 × 0.08 mm
*Z* = 4	

### Data collection

**Table:**

Rigaku Saturn CCD diffractometer	1928 independent reflections
Radiation source: fine-focus sealed tube	1642 reflections with *I* > 2σ(*I*)
graphite	*R*_int_ = 0.033
Detector resolution: 14.63 pixels mm^-1^	θ_max_ = 27.8°, θ_min_ = 2.6°
ω and φ scans	*h* = −10→10
Absorption correction: multi-scan (*CrystalClear*; Rigaku/MSC, 2005)	*k* = −20→21
*T*_min_ = 0.891, *T*_max_ = 0.954	*l* = −8→8
8141 measured reflections	

### Refinement

**Table:**

Refinement on *F*^2^	Primary atom site location: structure-invariant direct methods
Least-squares matrix: full	Secondary atom site location: difference Fourier map
*R*[*F*^2^ > 2σ(*F*^2^)] = 0.028	Hydrogen site location: inferred from neighbouring sites
*wR*(*F*^2^) = 0.076	H atoms treated by a mixture of independent and constrained refinement
*S* = 1.06	*w* = 1/[σ^2^(*F*_o_^2^) + (0.0461*P*)^2^] where *P* = (*F*_o_^2^ + 2*F*_c_^2^)/3
1928 reflections	(Δ/σ)_max_ < 0.001
113 parameters	Δρ_max_ = 0.44 e Å^−^^3^
1 restraint	Δρ_min_ = −0.21 e Å^−^^3^

### Special details

**Table:**

Geometry. All e.s.d.'s (except the e.s.d. in the dihedral angle between two l.s. planes) are estimated using the full covariance matrix. The cell e.s.d.'s are taken into account individually in the estimation of e.s.d.'s in distances, angles and torsion angles; correlations between e.s.d.'s in cell parameters are only used when they are defined by crystal symmetry. An approximate (isotropic) treatment of cell e.s.d.'s is used for estimating e.s.d.'s involving l.s. planes.
Refinement. Refinement of *F*^2^ against ALL reflections. The weighted *R*-factor *wR* and goodness of fit *S* are based on *F*^2^, conventional *R*-factors *R* are based on *F*, with *F* set to zero for negative *F*^2^. The threshold expression of *F*^2^ > σ(*F*^2^) is used only for calculating *R*-factors(gt) *etc*. and is not relevant to the choice of reflections for refinement. *R*-factors based on *F*^2^ are statistically about twice as large as those based on *F*, and *R*- factors based on ALL data will be even larger.

### Fractional atomic coordinates and isotropic or equivalent isotropic displacement parameters (Å^2^)

**Table:**

	*x*	*y*	*z*	*U*_iso_*/*U*_eq_	
S1	0.40446 (4)	0.02171 (2)	0.20702 (6)	0.01704 (11)	
S2	0.60139 (5)	−0.14291 (2)	0.26909 (6)	0.02389 (13)	
N1	0.27180 (16)	−0.11033 (7)	0.30185 (19)	0.0159 (3)	
N2	0.13949 (15)	−0.05360 (7)	0.28751 (18)	0.0151 (2)	
N3	−0.14570 (15)	0.23252 (7)	0.15350 (18)	0.0154 (3)	
C1	0.42543 (17)	−0.08492 (8)	0.2646 (2)	0.0159 (3)	
C2	0.19024 (17)	0.01952 (8)	0.2396 (2)	0.0138 (3)	
C3	0.07477 (17)	0.09310 (8)	0.2109 (2)	0.0138 (3)	
C4	0.13479 (18)	0.17060 (8)	0.1615 (2)	0.0160 (3)	
H4	0.2515	0.1772	0.1463	0.019*	
C5	0.02020 (18)	0.23800 (8)	0.1349 (2)	0.0159 (3)	
H5	0.0617	0.2909	0.1018	0.019*	
C6	−0.20252 (18)	0.15758 (8)	0.2012 (2)	0.0156 (3)	
H6	−0.3202	0.1529	0.2145	0.019*	
C7	−0.09795 (17)	0.08668 (9)	0.2320 (2)	0.0156 (3)	
H7	−0.1426	0.0348	0.2667	0.019*	
H1	0.244 (3)	−0.1638 (6)	0.325 (3)	0.047 (6)*	

### Atomic displacement parameters (Å^2^)

**Table:**

	*U*^11^	*U*^22^	*U*^33^	*U*^12^	*U*^13^	*U*^23^
S1	0.01417 (18)	0.01237 (18)	0.0254 (2)	−0.00003 (12)	0.00638 (15)	0.00162 (13)
S2	0.0190 (2)	0.0213 (2)	0.0318 (3)	0.00798 (14)	0.00682 (17)	0.00402 (15)
N1	0.0171 (6)	0.0109 (5)	0.0204 (7)	0.0014 (4)	0.0058 (5)	0.0016 (5)
N2	0.0171 (6)	0.0117 (5)	0.0170 (6)	0.0013 (5)	0.0048 (5)	0.0001 (4)
N3	0.0164 (6)	0.0132 (6)	0.0158 (6)	−0.0001 (5)	0.0021 (5)	−0.0016 (4)
C1	0.0169 (7)	0.0139 (6)	0.0162 (7)	0.0003 (5)	0.0025 (5)	−0.0005 (5)
C2	0.0142 (6)	0.0137 (6)	0.0137 (7)	−0.0005 (5)	0.0039 (5)	−0.0014 (5)
C3	0.0160 (7)	0.0136 (6)	0.0115 (7)	0.0002 (5)	0.0026 (5)	−0.0014 (5)
C4	0.0146 (7)	0.0158 (7)	0.0179 (7)	−0.0014 (5)	0.0046 (6)	−0.0002 (5)
C5	0.0183 (7)	0.0123 (6)	0.0167 (7)	−0.0022 (5)	0.0037 (6)	0.0000 (5)
C6	0.0142 (6)	0.0170 (7)	0.0158 (7)	−0.0015 (5)	0.0040 (6)	−0.0017 (5)
C7	0.0177 (7)	0.0126 (7)	0.0164 (7)	−0.0025 (5)	0.0042 (6)	−0.0006 (5)

### Geometric parameters (Å, °)

**Table:**

S1—C2	1.7437 (15)	C2—C3	1.4679 (18)
S1—C1	1.7452 (15)	C3—C4	1.3916 (19)
S2—C1	1.6555 (14)	C3—C7	1.3974 (18)
N1—C1	1.3485 (18)	C4—C5	1.3862 (19)
N1—N2	1.3631 (16)	C4—H4	0.9500
N1—H1	0.902 (9)	C5—H5	0.9500
N2—C2	1.2980 (17)	C6—C7	1.3844 (19)
N3—C5	1.3378 (18)	C6—H6	0.9500
N3—C6	1.3417 (17)	C7—H7	0.9500
			
C2—S1—C1	89.77 (6)	C7—C3—C2	120.65 (12)
C1—N1—N2	118.98 (11)	C5—C4—C3	118.43 (13)
C1—N1—H1	125.1 (12)	C5—C4—H4	120.8
N2—N1—H1	115.7 (13)	C3—C4—H4	120.8
C2—N2—N1	110.06 (11)	N3—C5—C4	123.48 (12)
C5—N3—C6	117.71 (11)	N3—C5—H5	118.3
N1—C1—S2	127.20 (11)	C4—C5—H5	118.3
N1—C1—S1	107.03 (10)	N3—C6—C7	123.17 (13)
S2—C1—S1	125.77 (8)	N3—C6—H6	118.4
N2—C2—C3	122.50 (12)	C7—C6—H6	118.4
N2—C2—S1	114.16 (10)	C6—C7—C3	118.57 (12)
C3—C2—S1	123.33 (10)	C6—C7—H7	120.7
C4—C3—C7	118.64 (12)	C3—C7—H7	120.7
C4—C3—C2	120.71 (12)		
			
C1—N1—N2—C2	−0.77 (17)	N2—C2—C3—C7	−0.8 (2)
N2—N1—C1—S2	−178.87 (10)	S1—C2—C3—C7	177.65 (11)
N2—N1—C1—S1	0.50 (15)	C7—C3—C4—C5	−0.1 (2)
C2—S1—C1—N1	−0.08 (10)	C2—C3—C4—C5	179.66 (12)
C2—S1—C1—S2	179.29 (11)	C6—N3—C5—C4	0.3 (2)
N1—N2—C2—C3	179.24 (12)	C3—C4—C5—N3	−0.3 (2)
N1—N2—C2—S1	0.65 (15)	C5—N3—C6—C7	0.1 (2)
C1—S1—C2—N2	−0.34 (11)	N3—C6—C7—C3	−0.5 (2)
C1—S1—C2—C3	−178.92 (12)	C4—C3—C7—C6	0.5 (2)
N2—C2—C3—C4	179.40 (13)	C2—C3—C7—C6	−179.28 (12)
S1—C2—C3—C4	−2.14 (19)		

### Hydrogen-bond geometry (Å, °)

**Table:**

*D*—H···*A*	*D*—H	H···*A*	*D*···*A*	*D*—H···*A*
N1—H1···N3^i^	0.90 (1)	1.85 (1)	2.7395 (19)	169 (2)

Symmetry codes: (i) −*x*, *y*−1/2, −*z*+1/2.

## References

[bb1] Liu, X. H., Chen, P. Q., Wang, B. L., Li, Y. H., Wang, S. H. & Li, Z. M. (2007). *Bioorg. Med. Chem. Lett.* **17**, 3784–3788. 10.1016/j.bmcl.2007.04.003 17512731

[bb2] Liu, X. H., Shi, Y. X., Ma, Y., He, G. R., Dong, W. L., Zhang, C. Y., Wang, B. L., Wang, S. H., Li, B. J. & Li, Z. M. (2009*a*). *Chem. Biol. Drug Des.* **73**, 320–327. 10.1111/j.1747-0285.2009.00779.x 19207468

[bb3] Liu, X. H., Shi, Y. X., Ma, Y., Zhang, C. Y., Dong, W. L., Li, P., Wang, B. L., Li, B. J. & Li, Z. M. (2009*b*). *Eur. J. Med. Chem.* **44**, 2782–2786. 10.1016/j.ejmech.2009.01.012 19246128

[bb4] Liu, X. H., Zhang, C. Y., Guo, W. C., Li, Y. H., Chen, P. Q., Wang, T., Dong, W. L., Sun, H. W. & Li, Z. M. (2009*c*). *J. Enzym. Inhib. Med. Chem.* **24**, 545–552. 10.1080/14756360802234943 18763167

[bb5] Rigaku/MSC (2005). *CrystalClear* Rigaku/MSC Inc., The Woodlands, Texas, USA.

[bb6] Sheldrick, G. M. (2008). *Acta Cryst.* A**64**, 112–122. 10.1107/S0108767307043930 18156677

[bb7] Song, B. A., Chen, C. J., Yang, S., Jin, L. H., Xue, W., Zhang, S. M., Zou, Z. H., Hu, D. Y. & Liu, G. (2005). *Acta Chem. Sin.* **18**, 1720–1726.

